# Editorial: Neuroimaging insights into the link between sleep disturbances and neuropsychiatric disorders

**DOI:** 10.3389/fpsyt.2023.1243486

**Published:** 2023-06-29

**Authors:** Karen M. von Deneen, Malgorzata A. Garstka, Tomáš Hrbáč, Yuanqiang Zhu, HuaNing Wang, Jun Chang Su

**Affiliations:** ^1^Center for Brain Imaging, School of Life Science and Technology, Xidian University, Xi'an, Shaanxi, China; ^2^International Joint Research Center for Advanced Medical Imaging and Intelligent Diagnosis and Treatment & Xi'an Key Laboratory of Intelligent Sensing and Regulation of Trans-Scale Life Information, School of Life Science and Technology, Xidian University, Xi'an, Shaanxi, China; ^3^Core Research Laboratory, Department of Endocrinology, Xi'an, Shaanxi, China; ^4^Department of Tumor and Immunology, Precision Medical Institute, Western China Science and Technology Innovation Port, School of Medicine, The Second Affiliated Hospital of Xi'an Jiaotong University, Xi'an, Shaanxi, China; ^5^Neurochirurgická Klinika, Fakultní Nemocnice Ostrava, Ostrava, Czechia; ^6^Department of Radiology, Xijing Hospital, Fourth Military Medical University, Xi'an, China; ^7^Xijing Hospital, Fourth Military Medical University, Xi'an, China; ^8^Department of Neurology, Tangdu Hospital, The Fourth Military Medical University, Xi'an, China

**Keywords:** sleep disorders, neuropsychiatric disorders, neuroimaging, cognition, memory, attention, sleep, brain

Sleep is a fundamental physiological process that plays a crucial role in maintaining overall health and wellbeing. Adequate and quality sleep is essential for cognitive functioning, emotional regulation, and various other aspects of human physiology. Sleep disturbances, on the other hand, have been associated with a wide range of neuropsychiatric disorders, including dementia, Parkinson's disease, major depressive disorder, bipolar disorder, post-traumatic stress disorder, attention deficit hyperactivity disorder, schizophrenia, and anxiety disorders. Despite significant research efforts, the underlying neural mechanisms of sleep, sleep disorders and the neurobiological consequences of sleep deprivation remain partially understood.

In recent years, neuroimaging modalities have provided unique insights into the neural correlates of sleep disturbances and neuropsychiatric disorders. Techniques such as electroencephalography (EEG), magnetoencephalography (MEG), functional magnetic resonance imaging (fMRI), and simultaneous EEG-fMRI studies have allowed researchers to examine the neural dynamics associated with sleep quality and quantity, as well as the structural and functional changes induced by sleep disturbances and neuropsychiatric diseases. These neuroimaging approaches have also facilitated investigations into how the brain adapts to these disorders and the potential consequences for mental health.

We are pleased that our special edition of this journal covers submissions predominantly from China, but also from Taiwan, Switzerland, Netherlands, Italy, Germany, UK, and USA. It brings together a Research Topic of articles that highlight the current state of research on sleep, sleep disturbances, and neuropsychiatric disorders using neuroimaging techniques. The studies cover a wide range of topics based upon the aims and objectives, including the mechanisms of sleep deprivation on cognitive functions and emotional state, the association between sleep disorders and depression, Alzheimer's disease, autism, and epilepsy, the impact of obstructive sleep apnea on mental health, the role of sleep spindles, the consequences of shift-work sleep disorders in the healthy population, and targeted treatments for insomnia and psychiatric diseases.

One area of research that has gained considerable attention is the impact of sleep disturbances on cognitive function and emotional regulation. Specific features of sleep, such as sleep spindles, have been found to be associated with memory consolidation and emotional processing. Neuroimaging findings revealed the neural mechanisms underlying these associations, shedding light on how sleep disturbances may contribute to cognitive and emotional impairments observed in various neuropsychiatric disorders. Patients with insomnia differed from healthy individuals in neural activity, and functional connectivity between the cholinergic and non-cholinergic sub-regions of the basal forebrain, suggesting that the basal forebrain plays an important role in sleep-wake regulation and may be implicated in cortical arousal and activation (Jiang et al.). Awakening was found to be accompanied by an increase in cerebrovascular reactivity and functional connectivity in the thalamus, insula, and primary motor cortex (Hsu et al.). Altered functional connectivity strength in patients with insomnia was associated with neuropsychological performance indicators and specific changes in gut microbiota composition, specifically genera *Alloprevotella, Lachnospiraceae*, and *Faecalicoccus* (Chen et al.). Sleep deprivation can lead to learning and memory impairment, reduced attention, and poor work performance. Individuals suffering from shift work disorder (SWD) displayed alterations in the fundamental architecture of interregional structural connectivity in the brain (Ning et al.). Sleep-deprived rats experienced weakened cognitive functions and memory that were related to impaired conventional protein kinase Cγ (cPKCγ)-neurogranin (Ng) signaling. Activation of the cPKCγ-Ng signal was shown to improve cognitive function and reduce memory impairment caused by sleep deprivation in rats (Xu et al.). Amygdala-based real-time fMRI neurofeedback training employed to treat insomnia patients reshaped neural activity and improved sleep quality in insomnia patients (Li Z. et al.).

Patients with insomnia are known to have a higher depression score, which was also seen in the studies in this Research Topic (Chen et al.; Jiang et al.). Two studies investigated pathophysiology of depression using fMRI. Guo X. et al. found that adolescents with depression had abnormal degree centrality in several brain regions, including the left superior temporal gyrus and right inferior parietal lobule. Patients with melancholic major depressive disorder with insomnia presented abnormal brain functional connectivity in multiple brain regions such as the right middle temporal gyrus, superior temporal gyrus, right middle occipital gyri, superior occipital gyri, right cuneus, bilateral lingual gyrus, and bilateral calcarine compared to healthy controls (Deng et al.). These studies highlight the potential of fMRI as a tool for identifying biomarkers of depression that may inform future research on treatment approaches for depression and could ultimately lead to more personalized and effective interventions. Indeed, fMRI was instrumental in identifying issues with neural activity in patients with depression and suicidal ideation (Tang et al.), postpartum depressive disorder (Zhang et al.), and late-life depression (Li H. et al.). Employing computational approaches, including network analysis and machine learning, the authors selected areas in the brain for treatment by target-transcranial magnetic stimulation (target-TMS) therapy that restored functional network connectivity, and allowed alleviation of depressive clinical symptoms (Tang et al.; Zhang Y. et al.). Brain connectivity in depression could also be improved with mindfulness-based cognitive therapy (MBCT). Patients with late-life depression who underwent MBCT experienced increased connectivity between the middle frontal gyrus and amygdala that corresponded to the reduction in depressive symptoms (Li H. et al.). TMS and MBCT are non-invasive and non-pharmacological interventions, and guided by neuroimaging, they could potentially relieve depression and improve the quality of life.

Besides depression, other psychiatric disorders are also accompanied by sleep disorders including epilepsy, Alzheimer's disease, schizophrenia, or autism. Guo M. et al. aimed to understand the relationship between temporal lobe epilepsy (TLE) and sleep disorders using MRI and diffusion kurtosis imaging. They found that TLE patients with sleep disorders had significantly higher mean diffusivity and radial diffusivity values in the left temporal lobe compared to TLE patients without sleep disorders. These findings suggest that there may be a link between TLE and sleep disorders, which could have implications for diagnosis and treatment.

In the systematic review, Liu et al. summarized studies that investigated structural and functional changes in the brain of Alzheimer's patients with sleep disorders. A range of imaging methods was reported, including MRI, single-photon emission computed tomography, multislice spiral computed tomography perfusion imaging, and 2-deoxy-2-(^18^F)fluoro-D-glucose positron emission tomography (^18^F-FDG-PET). They summarized that sleep disorders are common in Alzheimer's patients and may contribute to cognitive decline and other symptoms. The review also highlights several brain regions that are affected by sleep disorders in Alzheimer's patients, including the hippocampus, amygdala, and prefrontal cortex. Zhang A. et al. constructed an fMRI-based whole brain connectivity network to understand the neural mechanisms underlying the core symptoms of autism spectrum disorder (ASD) across different age groups. They identified that functional connectivity (FC) between the left superior occipital lobe and right angular, and the left insula and left caudate was related to the restricted and repetitive behaviors in individuals with ASD, and the decrease in FC between these regions coincided with improvement in autism symptoms.

Dimitrades et al. investigated the differences in sleep spindles between healthy youth and those with schizophrenia or anti-NMDA receptor encephalitis using EEG. They found that patients with schizophrenia or anti-NMDA receptor encephalitis had reduced spindle density and duration compared to healthy young adults. As many individuals with Alzheimer's disease, autism (1), or schizophrenia (2) experience sleep disorders, the findings of these studies should be further explored to address disrupted sleep.

Sleep disturbances are often also seen in other disorders, including hypertension, obesity, type 2 diabetes mellitus, or cardiovascular disease. Neuroimaging methods could be used to reveal the neural mechanisms underlying these associations, and shed light on sleep disturbances in these conditions (3), while computational approaches could enable the identification of distinct subtypes of sleep impairment and provide opportunities for personalized interventions in patients.

## Author contributions

All authors listed have made a substantial, direct, and intellectual contribution to the work and approved it for publication.
